# Mind the Anion Gap: 5-Oxoproline-Induced High Anion Gap Metabolic Acidosis in End-Stage Renal Disease

**DOI:** 10.7759/cureus.61328

**Published:** 2024-05-29

**Authors:** Danny Kwon, Michelle Seu, Sylvester Barnes

**Affiliations:** 1 Department of Internal Medicine, Loyola University Medical Center, Maywood, USA; 2 Department of Neurology, Loyola University Medical Center, Maywood, USA; 3 Department of Nephrology, Loyola University Medical Center, Maywood, USA

**Keywords:** glutathione, renal replacement therapy (rrt), end stage renal disease (esrd), acetaminophen, 5-oxoproline, high anion gap metabolic acidosis

## Abstract

A rare complication, 5-oxoproline-induced high anion gap metabolic acidosis (HAGMA) is associated with chronic acetaminophen use, predominantly reported in outpatient settings. However, its occurrence in hospitalized patients, particularly those with end-stage renal disease (ESRD), remains underreported. We present a case of a 74-year-old female with ESRD on hemodialysis who developed HAGMA highly suspicious for 5-oxoproline toxicity from acetaminophen usage following cardiac surgery. Despite a standard analgesic dose, the patient's renal impairment likely predisposed her to 5-oxoproline accumulation, resulting in severe metabolic acidosis. Discontinuation of acetaminophen led to the resolution of HAGMA, highlighting the importance of recognizing this rare but potentially life-threatening complication in the inpatient and critical care setting. This case suggests a potential interaction between acetaminophen metabolism and renal dysfunction in the pathogenesis of 5-oxoproline-induced HAGMA.

## Introduction

Pyroglutamic acid or 5-oxoproline is a rare cause of high anion gap metabolic acidosis (HAGMA) that can manifest in individuals with a history of chronic acetaminophen ingestion. It is believed that acetaminophen may deplete glutathione, potentially through the inhibition of glutathione synthetase by a toxic metabolite known as N-acetyl-p-benzoquinone imine (NAPQI), thereby leading to the accumulation of 5-oxoproline in the bloodstream [1].

While most cases of 5-oxoproline-induced HAGMA have been documented among chronically ill and malnourished women with a history of chronic acetaminophen use, the levels of acetaminophen detected in these cases are typically not in the toxic range [2,3]. It is conceivable that numerous additional cases have either gone unreported or remained undiagnosed due to limited awareness of this condition and restricted availability of 5-oxoproline urine or serum assays [4].

Here, we present a case of a woman with end-stage renal disease (ESRD) on hemodialysis, who developed 5-oxoproline-induced HAGMA from a postoperative acetaminophen regimen.

## Case presentation

A 74-year-old female with a significant medical history of hypertension, systemic lupus erythematosus, and ESRD, undergoing intermittent hemodialysis for 13 years, was admitted for coronary artery bypass graft (CABG) and aortic valve replacement. Prior to admission, she developed a sizable hematoma over the arteriovenous fistula (AVF), necessitating the temporary placement of a right femoral dialysis catheter. In the outpatient setting, the AVF remained her primary dialysis access, with the femoral catheter retained for potential postoperative continuous renal replacement therapy. Her outpatient medications included acetaminophen with codeine 15-300 mg as needed, amlodipine 5 mg daily, apixaban 5 mg twice daily, aspirin 81 mg daily, carvedilol 3.125 mg twice daily, gabapentin 100 mg three times daily, levothyroxine 88 mcg daily, omeprazole 20 mg daily, prochlorperazine 5 mg as needed, and sevelamer 800 mg with meals.

During her inpatient stay, she underwent three-vessel coronary artery bypass grafting with on-pump bypass, aortic valve replacement, and tricuspid valve repair. Subsequently, she was admitted to the cardiovascular intensive care unit (CVICU) for postoperative care. Nephrology consultation occurred on postoperative day (POD) 1, with her last dialysis session being two days prior. Inpatient medications included acetaminophen 650 mg every six hours, aspirin 325 mg daily, atorvastatin 40 mg daily, gabapentin 100 mg three times daily, heparin 5000 units every eight hours, hydroxychloroquine 200 mg every 12 hours, levothyroxine 88 mcg daily, pantoprazole 40 mg daily, polyethylene glycol 17 g daily, and potassium chloride administered 20 mEq as needed. Hemodialysis resumed on POD1 without ultrafiltration.

Later in the evening of POD1, the CVICU team requested aggressive fluid removal, leading to the initiation of continuous venovenous hemofiltration (CVVH). Her laboratory values at this time showed no significant abnormalities (Table 1). Over the next two weeks, she developed progressively worsening altered mental status and dysphagia, necessitating tracheostomy as well as percutaneous endoscopic gastrostomy (PEG) tube placement on POD14. Additional postoperative complications included *Enterococcus* bacteremia, treated with ampicillin and cefepime, followed by septic shock necessitating norepinephrine and vasopressin infusion for hemodynamic support.

**Table 1:** Serum electrolytes on the evening of postoperative day 1

Test	Result	Reference range
Sodium	135 mEq/L	135-145 mEq/L
Potassium	4.6 mEq/L	3.5-5.0 mEq/L
Chloride	99 mEq/L	95-105 mEq/L
Bicarbonate	25 mmol/L	22-33 mmol/L
Blood urea nitrogen	22 mg/dL	8-20 mg/dL
Creatinine	3.02 mg/dL	0.6-1.5 mg/dL
Glucose	88 mg/dL	70-110 mg/dL
Calcium	9.6 mg/dL	8.5-10.5 mg/dL
Phosphorous	4.5 mg/dL	2.0-4.5 mg/dL

Due to hemodynamic instability and increasing pressor requirements, CVVH was continued until POD26. On POD26, she transitioned to intermittent renal replacement therapy (AVVH) for eight hours in the morning, followed by slow continuous ultrafiltration (SCUF). However, on the same evening, she developed high anion gap metabolic acidosis (HAGMA) with a bicarbonate level of 16 mmol/L. An arterial blood gas (ABG) performed at the same time confirmed the presence of acidosis, with a relatively normal partial pressure of carbon dioxide (PCO_2_) (Table 2). Lactic acid concentration was also insignificant. Due to significant acidosis, CVVH was restarted on POD27.

**Table 2:** Arterial blood gas and lactic acid on the evening of postoperative day 26 PCO_2_: partial pressure of carbon dioxide; PO_2_: partial pressure of oxygen; HCO_3_: bicarbonate

Test	Result	Reference range
pH	7.29	7.35-7.45
PCO_2_	34 mm Hg	35-45 mm Hg
PO_2_	183 mm Hg	80-100 mm Hg
HCO_3_	16 mmol/L	22-26 mmol/L
Lactic acid	0.8 mmol/L	0.5-1.6 mmol/L

At that time, several potential etiologies for the patient's HAGMA were considered. Although she had recently started tube feeds with insulin, glucose levels were maintained at 111-152 mg/dL with low concern for ketones as the source of anions. A D-lactic acid level was sent for possible bacterial gut overgrowth, but the result was unremarkable. A comprehensive review of the medication regimen revealed scheduled acetaminophen since surgery 29 days ago. With common culprits of HAGMA ruled out, accumulation of 5-oxoproline due to acetaminophen was postulated. Although 5-oxoproline levels were not measured, acetaminophen was discontinued on POD29 given high clinical suspicion.

On the evening of POD30, CVVH was again discontinued with intermittent hemodialysis started on the morning of POD31. At that time, the patient transitioned back to a three-times-per-week hemodialysis schedule with her next hemodialysis scheduled for POD33. By POD33, after holding renal replacement therapy for over 48 hours and discontinuing acetaminophen four days prior, her bicarbonate level reached a nadir of 21 mmol/L, marking the resolution of HAGMA to a normalized level of acidosis in the setting of her ESRD.

The remainder of her hospital course included a transesophageal echocardiogram on POD49 showing significant improvement in cardiac function, fistulogram and fistuloplasty for AVF on POD57, and transfer to a long-term acute care hospital on POD59.

## Discussion

in 1989, 5-oxoproline was first noted as a cause of anion gap metabolic acidosis by Creer et al. This likely occurred in the setting of acetaminophen ingestion; however, the authors did not commit to this as the likely etiology at the time [5,6]. In the following year, Pitt et al. described another case of 5-oxoproline metabolic acidosis and proposed the link between this ailment and chronic acetaminophen ingestion [6,7]. Since this period, chronic acetaminophen use remains an uncommon but better-recognized cause of HAGMA, with various case reports reflecting this [6-8]. Most cases of 5-oxoproline-induced HAGMA pertain to female patients with various chronic medical conditions, malnutrition, and frequent acetaminophen use in the outpatient setting [6-9]. Interestingly, it has been less commonly reported with inpatient acetaminophen use [6].

Prior pharmacokinetic studies have shown that acetaminophen is extensively metabolized, with only around 3% of the drug excreted in its original form [10,11]. Most metabolites of acetaminophen are sulfate and glucuronide conjugates, while a smaller component of acetaminophen is metabolized to a highly reactive alkylating metabolite known as N-acetyl-p-benzoquinone imine (NAPQI) [10]. NAPQI is a well-known culprit of hepatic necrosis in the setting of acetaminophen excess, but at normal therapeutic concentrations of acetaminophen, NAPQI is typically inactivated by intracellular glutathione and subsequently excreted in urine [10,11]. This partially explains why our patient with significant renal impairment with no urine production was at a higher risk of accumulating toxic levels of conjugated acetaminophen metabolites, even at normal therapeutic dosages. Hemodialysis can be used to rapidly decrease the serum concentration of acetaminophen to prevent further production of NAPQI, but there are no current data to suggest that NAPQI is also dialyzable [12].

**Figure 1:**
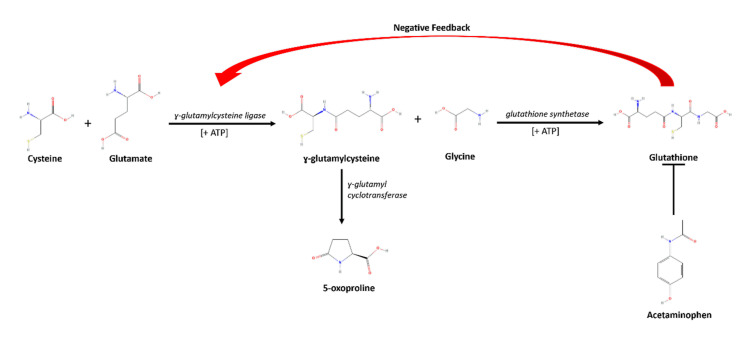
General mechanism of 5-oxoproline toxicity in the setting of acetaminophen usage Image Credit:  Michelle Seu

The risk of 5-oxoproline accumulation and HAGMA is higher in certain individuals who either endogenously lack glutathione synthase or acquire glutathione deficiency due to other comorbidities. This is because the primary role of glutathione is to protect cells from both naturally occurring and exogenous reactive molecules by reducing and consequently inactivating them [9,13]. Glutathione is made from the enzymatic combination of the amino acids cysteine and glutamate to produce g-glutamylcysteine via glutamate cysteine ligase [9]. Individuals who have deficiencies or absence of glutathione synthase suffer from 5-oxoprolinuria and acidosis [14]. Furthermore, without appropriate feedback inhibition, glutamate cysteine ligase will continue to produce g-glutamylcysteine, leading to the eventual overproduction of 5-oxoproline by the enzyme g-glutamyl cyclotransferase, which overwhelms its associated degradative enzyme 5-oxoprolinase (Figure 1) [14]. The same feedback loop dysfunction and acquired lack of the amino acids cysteine, methionine, and glycine explain why 5-oxoproline accumulation tends to occur in the setting of renal impairment, sepsis, and poor nutritional status [9].

## Conclusions

This report details the case of an elderly woman with ESRD undergoing hemodialysis, who developed various complications following cardiac surgery, including the suspected 5-oxoproline-induced HAGMA. The HAGMA swiftly resolved upon discontinuation of her postoperative acetaminophen regimen. This case report is a valuable addition to the literature, addressing the scarcity of reported 5-oxoproline-induced HAGMA in the inpatient setting. Furthermore, it underscores the vulnerability of patients with severe renal impairment and malnutrition to developing conditions such as HAGMA.
